# Burn Injuries at Jordan University Hospital: A Five-Year Retrospective Study with Historical Comparison

**DOI:** 10.3390/healthcare14111473

**Published:** 2026-05-26

**Authors:** Bareqa Salah, Mohammad Al-Hanaktah, Ehab Alroud, Omar Awadallah, Mahmoud Shehabat, Ahmad AL-Qunbar

**Affiliations:** 1 Department of General Surgery/Plastic & Reconstructive, Faculty of Medicine, University of Jordan, Amman 11942, Jordan; 2 Faculty of Medicine, University of Jordan, Amman 11942, Jordan; malhanaktah49@gmail.com (M.A.-H.); ehabroud@gmail.com (E.A.); mahmoudshehabat99@gmail.com (M.S.); qqunbar2003@gmail.com (A.A.-Q.); 3 Department of General Surgery, Faculty of Medicine, University of Jordan, Amman 11942, Jordan; omarawadallah2011@gmail.com

**Keywords:** burn units, burns, burden, demographics, retrospective studies, Jordan

## Abstract

**Background**: Burn injuries remain a major health problem in low- and middle-income countries. Contemporary data from Jordan are scarce, and the last report from Jordan University Hospital (JUH) was published more than four decades ago. This study describes the epidemiology, characteristics, management, and outcomes of burn admissions to JUH during 2016–2020 and compares them with historical and regional data. **Methods**: We conducted a retrospective cohort study of all consecutive patients admitted to the JUH burn unit with acute burn injury between 1 January 2016 and 31 December 2020. Demographic and clinical variables were abstracted from electronic and paper records using a standardised case-report form. Descriptive statistics summarised injury patterns, while bivariate tests and multivariable linear regression were used to identify factors associated with hospital length of stay (LOS). **Results**: A total of 575 patients were included (50.3% male; median age 19 years). Children constituted 43.5% of admissions. Median TBSA was 7%, and partial-thickness burns predominated (73.9%). Scalds were the leading aetiology (60.7%), followed by flame burns (19.5%). Most injuries occurred at home (92.5%). The median LOS was 6 days, and 2.1% of patients died. Burn degree, aetiology, TBSA, surgical grafting, and adverse clinical events were independently associated with longer LOS, whereas escharectomy shortened hospitalisation. **Conclusions**: Domestic scald injuries in children remain the dominant burn pattern in Jordan, although mortality has fallen markedly compared with the 1980 JUH cohort. Prevention efforts should prioritise household safety and child supervision, while continued investment in specialised burn care is likely to further improve outcomes.

## 1. Introduction

Burns represent a significant global health challenge, with the World Health Organization estimating over 180,000 deaths annually from fire-related injuries alone [1]. The Global Burden of Disease Study 2019 reported approximately 8.95 million new burn cases globally, with age-standardised incidence rates of 117.5 per 100,000 population [2]. The burden disproportionately affects low- and middle-income countries (LMICs), where more than 90% of burn-related deaths occur, as reported by the analysis of Peck (2011) [3]. Subsequent registry- and review-based syntheses have shown persistence during the past decade [4,5].

The Eastern Mediterranean Region (EMR), as defined by the World Health Organization (WHO), comprises 22 nations, including Jordan, and exhibits distinct patterns of burn epidemiology. A systematic review conducted by Othman and Kendrick (2010), which analysed 71 studies from 12 EMR countries, reported burn incidence rates ranging from 112 to 518 per 100,000 per year, with hospital mortality between 5 and 37% [6]. This review illustrated several regional features: a predominance of young victims (mean age 18–25 years), high rates of pediatric burns (up to 38% in children aged 0–5 years), female predominance in several nations, and concerning rates of intentional self-harm burns among females in certain countries [6].

Further EMR data revealed that flame burns were the predominant cause among hospitalised patients, often associated with higher mortality compared to scalds [6]. The majority of injuries occurred at home (72–94%) and demonstrated a seasonal peak in winter months, accounting for 28–44% of cases [6]. Hospitalised patients showed a wide range of total body surface area (TBSA) involvement, ranging from 10 to 48% [6]. Country-specific studies highlighted these variations. In Kuwait, a review of 1702 cases showed a mean TBSA of 10% and a mortality rate of 5.3%, with the highest incidence among children aged 0–4 years [7]. In Iran, a study from Kurdistan province documented an age-standardised incidence rate (ASIR) of 149.86 per 100,000, with flame burns predominating, a mean TBSA reaching 48%, and a mortality of 33%, largely due to intentional self-harm [8]. In Saudi Arabia, paediatric data revealed scalds as the leading cause of burns in children, with a mean TBSA of 15% and a 2.8% mortality rate [9].

In the context of the growing burden of burns in Jordan, available epidemiological data remain limited. One of the earliest reports, a retrospective review of 338 admissions to Jordan University Hospital between 1976 and 1980, found that flame burns (53%) and scalds (42%) were the leading causes of burns, with children accounting for more than half of cases, particularly those under five years [10]. Most injuries occurred at home (88%), often linked to unsafe kitchen and heating practices, and males were more frequently affected [10]. Mortality reached 11.8%, largely due to septicaemia and hypovolemic shock, with outcomes worsened by the absence of a dedicated burns unit [10].

A more recent cross-sectional study of 193 patients from hospitals in the centre north of Jordan reported a predominance of scald injuries (50.5%), most of which occurred at home (88.6%), with male predominance (52.8%), and children aged 1–10 years as the most affected group (35.2%) [11]. While providing updated insights, the study was limited by its small sample size, short duration, and lack of outcome data.

Collectively, these findings highlight burns as a significant public health burden in the EMR and emphasise the need for country-specific evaluations, particularly in Jordan, where data remain limited. This study aims to describe the epidemiological characteristics, clinical patterns, and outcomes of burn injuries treated at Jordan University Hospital (JUH) over five years and compare them with the previous study conducted at JUH during the period 1976–1980, as well as with regional and global data.

## 2. Materials and Methods

### 2.1. Study Design, Setting, and Period

We conducted a retrospective cohort study at Jordan University Hospital (JUH), a tertiary academic medical centre in Amman, Jordan, serving as a referral site and the first site patients visit for multiple conditions, including burn care. The study period spanned 1 January 2016 to 31 December 2020. The protocol, variables, and analysis plan were defined in the institutional research proposal before the study was conducted.

### 2.2. Participants and Eligibility Criteria

Our study targeted all patients of any age admitted to the burn unit at JUH for management of acute burn injury during the study period.

Inclusion criteria. (i) Hospital admission to the burn unit for an acute burn; (ii) burn mechanism of thermal (flame, scald, contact), chemical, electrical, radiation, or friction; (iii) availability of a medical record with core variables (the medical record contained the prespecified minimum core dataset—age, sex, date of injury, date of admission, date of discharge, burn mechanism, and TBSA). Records that were retrievable but lacked any one of these core variables were excluded.

Exclusion criteria. (i) Patients with completely missing medical records; (ii) sunburn or isolated minor first-degree burns not requiring admission; (iii) patients transferred out immediately without receiving definitive care at JUH; (iv) injuries occurring outside the study window.

### 2.3. Case Ascertainment and Data Sources

Cases were identified from JUH’s hospital information system using ICD-10 diagnostic codes T20–T32 (burns and corrosions) and external cause codes X00–X19 (exposure to smoke, fire, flames, heat, and hot substances), cross-referenced against burn unit admission analogue logs, emergency department (ED) disposition records, operating room registries, and ICU census lists to ensure completeness. For eligible admissions, data were abstracted from the electronic medical record (and paper charts where applicable) using a standardised case report form (CRF). Cases were collected on a consecutive basis.

### 2.4. Variables and Operational Definitions

#### 2.4.1. Demographics

Age was recorded in completed years at the date of injury and was analysed as a continuous variable; for stratified analyses, we additionally derived a binary “age group (2-cat)” variable, with “child” defined as ≤18 years and “adult” as >18 years, matching paediatric thresholds used in the WHO Global Burn Registry. Sex was recorded as biological sex (male/female) from the patient record. Comorbidities were abstracted from the admission documentation and dichotomised as “none” vs. “any” for the bivariate tables; the underlying conditions (e.g., diabetes mellitus, hypertension, cardiovascular disease, chronic kidney disease, psychiatric history, etc.) were retained in the source dataset for sensitivity analysis. Occupation was coded into eight mutually exclusive categories from the patient’s documented social history: preschooler (not yet enrolled in school), student, government employee, private-sector employee, freelancer/self-employed, housewife, retired, and unemployed. Nationality was recorded as Jordanian vs non-Jordanian. Residence was recorded as the patient’s governorate of habitual residence at the time of injury (Amman, Balqa, Zarqa, Madaba, Irbid, Jerash, Ajloun, Mafraq, Karak, Tafilah, Ma’an, or Aqaba).

#### 2.4.2. Characteristics of Injury

- Mechanism: Thermal (flame, scald, contact), chemical (acid, alkali, other), electrical (high/low voltage), radiation, friction/other.
- Burn extent (TBSA): Percentage of total body surface area estimated clinically (rule of nines), recorded as continuous.
- Burn Degree: Superficial/(1st degree), partial-thickness (2nd degree, including superficial/deep), and full-thickness (3rd degree).
- Anatomical sites: Head/neck, trunk, upper/lower extremities, perineum.
- Admission and discharge dates: Calculated as length of stay (LOS)
- Context: Place of injury (home, workplace, public/other); date and time of injury; time to presentation.
- Treatment modalities: Conservative management, operative procedures (debridement, grafting, reconstruction), antimicrobial therapy, nutrition support, ICU admission and ventilation, and blood product utilisation.
- Death: If due to the injury, and its direct cause.

#### 2.4.3. Historical Comparator

To contextualise contemporary findings, we prespecified a structured *institution-level* comparison with the 1976–1980 JUH cohort published by El-Muhtaseb and colleagues [10]. We emphasise that this is not a patient-level pooled analysis. Patient-level data are not available for the historical cohort, and the two cohorts were assembled using different case-ascertainment methods—the 1976–1980 cohort from contemporaneous paper-based clinical records and the present cohort from ICD-10-coded electronic records cross-referenced with multiple operational logs (Section 2.3). Accordingly, the comparison is conducted exclusively at the level of published summary statistics (proportions, medians/means, and category distributions), using harmonised operational definitions wherever the two record systems permit. We do not perform inferential statistical tests across the two cohorts; reported differences are descriptive in nature and are interpreted in the context of differing case-ascertainment infrastructures, which is acknowledged as a primary limitation.

### 2.5. Data Collection, Management, and Quality Assurance

Data collectors were trained on definitions and CRF completion—a detailed data dictionary governed variable entry and coding. Double data entry was performed for a random 20% sample with reconciliation of discrepancies; periodic logic checks (range checks for TBSA 0–100%, mutually exclusive site coding, chronological plausibility of injury → presentation → admission) were run before dataset lock. Records were de-identified and stored in a secure, access-controlled database.

### 2.6. Statistical Analysis

Analyses followed a prespecified plan aligned with STROBE recommendations. Analyses were performed in R (v4.3) and SPSS (v28). Continuous variables are reported as median (IQR), as appropriate (not all variables were normally distributed), and categorical variables as counts and percentages. Temporal patterns were explored by year and season. Regarding bivariate analyses, between-group comparisons used χ^2^ or exact tests for categorical variables and the Kruskal–Wallis test for ≥3 groups. Post hoc Mann–Whitney tests were conducted with a Holm–Bonferroni adjustment. Normality was assessed using Shapiro–Wilk tests and visual diagnostics.

#### 2.6.1. Multivariable Models

Hospital LOS (our primary outcome): We fit a single multivariable (multiple) linear regression model with hospital LOS (days) as the dependent variable. Covariates were specified a priori on clinical and literature grounds and entered into the model by *forced entry*; no stepwise, forward, or backward selection procedure was used, to avoid the well-recognised instability and Type I error inflation associated with data-driven variable selection in observational data of this size. Model diagnostics included visual inspection of residual-versus-fitted plots (linearity, homoscedasticity), Q–Q plots of residuals (normality), and Cook’s distance (influential observations). Collinearity was assessed by the Variance Inflation Factor (VIF) and tolerance for every predictor; all predictors had VIF < 5 and tolerance > 0.20; thus, no covariate was removed on collinearity grounds. Because LOS and TBSA were right-skewed (Shapiro–Wilk *p* < 0.001), we retained the original metrics in the primary model to maintain coefficient interpretability (days and per cent TBSA) and used heteroskedasticity-consistent (HC3) robust standard errors. As a sensitivity analysis, we re-fit the model using log(LOS + 1) as the outcome; the direction and statistical significance of all major predictors were preserved. Coefficients for rare adverse events (atrial fibrillation, aphasia, end-stage kidney disease, reactive thrombocytosis) are derived from very small subgroups and are, accordingly, presented with wide confidence intervals; we interpret them descriptively rather than as stable causal effect estimates. A pre-specified sensitivity model in which these rare events were collapsed into a single binary composite (“any serious in-hospital adverse event”) yielded consistent overall conclusions.

#### 2.6.2. Bias, Confounding, and Sensitivity Analyses

We addressed potential selection bias by including all eligible admissions over the five-year census. Information bias was mitigated by standardised CRFs, abstractor training, and cross-validation of key variables (e.g., TBSA, LOS) across clinical notes, operative reports, and burn unit records. Confounding was addressed through multivariable adjustment informed by clinical knowledge and literature. Sensitivity analyses were included.

#### 2.6.3. Ethical Considerations and Reporting

The study protocol received approval from the Institutional Review Board (IRB) of Jordan University Hospital (Reference Number 10/2025/30829, dated 18 November 2025) and the JUH Scientific Committee, with a waiver of informed consent owing to the fully retrospective design and minimal risk to participants.

## 3. Results

**Figure 1:**
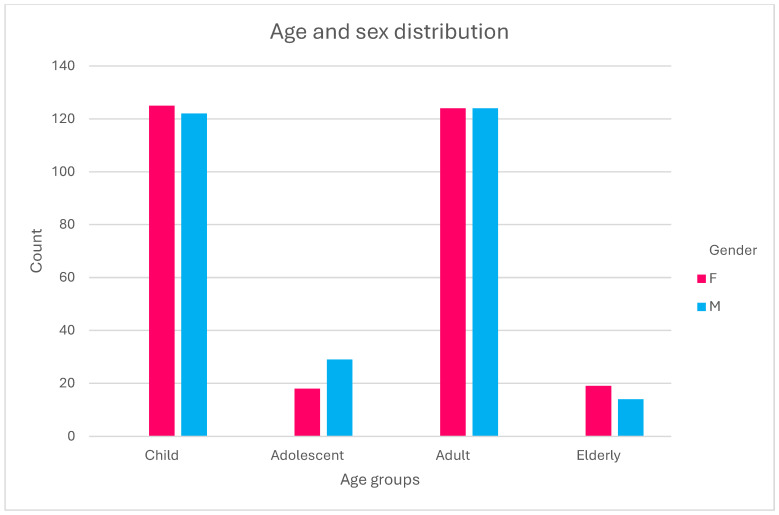
Burn distribution by age group and sex.

A total of 575 patients with burn injuries were included in the analysis. The cohort comprised 289 males (50.3%) and 286 females (49.7%), with a median age of 19 years (IQR 37). Children accounted for 43.5% of cases (n = 287). The vast majority of patients were Jordanian nationals (94.8%) (Table 1) (Figure 1).

**Figure 2:**
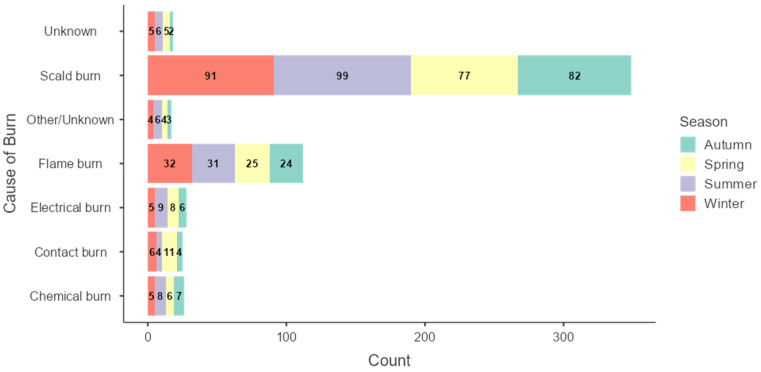
Seasonal distribution by type of burn.

Partial-thickness burns were the most common, affecting 73.9% of patients (n = 425), followed by full-thickness burns (13.0%, n = 75) and superficial burns (2.1%). The overall median TBSA was 7% (IQR 5.32). Regarding aetiology, scald burns were the most prevalent (60.7%), followed by flame burns (19.5%), electrical burns (4.9%), and chemical burns (4.5%). Contact and other causes together accounted for less than 10% of cases. The most frequently affected anatomical regions were multiple sites (32.0%) and the lower limbs (19.8%) (Table 2) Figure 2.

Most injuries occurred at home (92.5%), while 3.5% occurred in the workplace and the remainder in other settings, such as kitchens, streets, or schools. Seasonal variation was not statistically significant (*p* = 0.258). Children (62.5%) and students (40.0%) accounted for a large proportion of scald-related injuries, reflecting the domestic context in which most burns occurred (Table 1).

Burn severity associations are fully detailed in Table 1. Clinically, full-thickness burns affected older patients (median age 27 vs 13 years for partial-thickness burns; *p* < 0.001), involved a larger TBSA (*p* < 0.001), required surgical grafting more frequently, and were associated with longer hospitalisation (*p* < 0.001). Electrical burns carried the highest proportion of full-thickness injury (*p* < 0.001). Partial-thickness burns were the predominant type across all age groups, aetiologies, and anatomical sites (*p* < 0.001) (Table 1).

Aetiology associations are detailed in Table 2. Scalds predominated in children (76.0% of paediatric vs. 45.5% of adult cases; *p* < 0.001) and home settings (*p* < 0.001). Flame and electrical burns were more common in adults, males, and non-Jordanian patients; electrical burns carried the highest full-thickness injury rate and the greatest TBSA. Flame burns most frequently required surgical grafting, while electrical and chemical burns were associated with the longest hospitalisation (*p* < 0.001) (Table 2).

Post hoc comparisons provided further detail. In terms of age, significant differences were observed between the electrical and scald, electrical and flame, electrical and unknown, chemical and scald, chemical and flame, and chemical and unknown groups. For length of stay, significant differences were noted between electrical and scald, electrical and flame, electrical and unknown, chemical and scald, chemical and flame, and chemical and unknown. Regarding TBSA, contact burns differed significantly from the scald, flame, electrical, and unknown categories. Chemical burns differed significantly from the flame, electrical, and unknown categories, and scald burns differed significantly from the flame and unknown categories (Table 2).

Predictors of length of hospital stay were further explored in a multiple linear regression model (Table 3). The model explained 29.9% of the variance (adjusted R^2^ = 0.263, F(28,546) = 8.31, *p* < 0.001). Several clinical and treatment variables were significant. Surgical grafting was associated with a longer stay (+14.6 days, *p* < 0.001), whereas escharotomy was associated with a shorter stay (–11.5 days, *p* = 0.002). Adverse clinical outcomes prolonged stay markedly, including mortality (+16.2 days, *p* < 0.001), atrial fibrillation (+44.1 days, *p* < 0.001), aphasia (+58.8 days, *p* < 0.001), end-stage kidney disease requiring dialysis (+53.5 days, *p* < 0.001), and reactive thrombocytosis (+35.9 days, *p* = 0.010). Geographic differences were also noted, where those who were lived in Al-Zarqa’a had longer hospitalisation than those who lived in Amman (+5.0 days, *p* = 0.024). TBSA was a strong predictor of prolonged stay (+32.1, *p* < 0.001). Burn degree remained significant, with partial-thickness (–8.7 days, *p* < 0.001), superficial (–9.5 days, *p* = 0.036), and unknown (–8.9 days, *p* < 0.001) burns associated with shorter hospitalisations compared to full-thickness burns. Burn aetiology also played a role, as flame (+7.5 days, *p* = 0.017) and scald burns (+6.1 days, *p* = 0.039) were associated with longer hospitalisations relative to chemical burns (Table 3).

In our study, a total of 12 patients were deceased, a mortality rate of 2.1%, with the most common cause of death being cardiac arrest (n = 7), followed by sepsis (n = 3), and acute kidney injury (n = 2).

## 4. Discussion

The present findings provide a contemporary profile of burn injuries at Jordan University Hospital and allow for a meaningful comparison with the earliest comprehensive report from the same institution published by Al-Muhtasib et al. (1980) [10] as well as with regional and global trends. While both studies share the same institutional context, the current analysis reflects a considerably expanded sample size (575 vs 338 patients) and a modernised healthcare system, enabling a valuable comparison spanning over four decades.

### 4.1. Demographic and Age Distribution

Al-Muhtasib et al. reported a strong male predominance (ratio > 2:1) and a concentration of cases among children under 15 years (54.5%), with one-third of the total sample below five years of age [10]. In contrast, our data show a near-equal sex distribution (50.3% males, 49.7% females) and a more balanced age composition, with children constituting 43.5% of all cases. This narrowing gender gap and relative decline in the paediatric proportion likely reflect shifts in household supervision, educational access, and women’s participation in the workforce, together with improved public health awareness of domestic burn risks [5,12,13].

The demographic profile in our study varied slightly in some characteristics. It also showed similarities with regional and global patterns [4,5,12,14,15]. The World Health Organization Global Burn Registry documented a similar sex distribution (62% male, 38% female), with a slight male predominance. In the Middle East and North Africa (MENA) meta-analysis that synthesised 101 studies, males comprised 58.6% (95% CI 56.9–60.2%) [16]. Furthermore, the relatively young median age at JUH is consistent with global burn epidemiology patterns, in which children frequently represent the largest affected demographic group [4,5,12,14,15].

The predominance of Jordanian nationals (94.8%) in our study reflects the JUH’s role as a primary referral centre serving the domestic population, and is comparable to patterns observed in other Jordanian burn centres [14,15,17,18]. This pattern contrasts with the Kuwaiti experience reported by Khashaba et al. [19], where non-Kuwaiti expatriate workers represented the larger share of burn admissions. In that setting, the demographic was dominated by working-age males engaged in construction, industrial, and domestic-service occupations, producing a different exposure spectrum weighted toward flame and occupational burns [19].

### 4.2. Burn Aetiology and Setting

Both the 1980 cohort and our study identified scalds and flame burns as the leading causes; however, their proportions and contexts have changed markedly. In the 1980 cohort, fire burns accounted for 53% of cases and scalds 42%. In the current study, scalds became predominant (60.7%), while flame burns decreased to 19.5% [10]. A plausible interpretation of this reversal—consistent with regional energy-transition data and global paediatric burn evidence—is improved control of open-flame exposure, a near-disappearance of kerosene stoves from Jordanian households, and greater reliance on electric and gas cooking technologies, set against the continued vulnerability of young children to hot-liquid accidents during routine domestic activities [20,21,22]. Electrical and chemical burns, rarely reported previously (13 and 4 cases respectively), now constitute 4.9% and 4.5%, mirroring modernisation, industrial expansion, and widespread electricity use [10].

Al-Muhtasib et al. observed that 88% of burns occurred at home, rising to 96% in children [10]. The current findings show a similar figure (92.5%), confirming the home as the dominant risk environment despite societal transformation [10]. Nevertheless, workplace-based burns accounted for 3.5% of admissions in our cohort. In the absence of a directly matched 1980 workplace denominator, we cannot quantify a temporal “rise” with certainty; however, the figure is consistent with the broader regional and international literature describing the emergence of occupational electrical, chemical, and flame injuries as workforce structures and energy use have modernised.

Scalds also dominated among burn victims internationally and across MENA; the MENA meta-analysis estimates the scald prevalence at 50%, with approximately 80% of injuries occurring at home [16]. Global syntheses similarly report scald predominance in paediatric cohorts and domestic settings as the modal context of injury [4,13,23].

However, the relatively high proportion of electrical burns (4.9%) at JUH exceeds global averages reported in many Western studies but aligns with patterns observed in other developing regions [24,25,26]. Studies from Indonesia and Cameroon documented electrical burn rates of 34.3% and 26.3%, respectively, suggesting that variations in infrastructure and occupational safety may contribute to regional differences in electrical burn incidence [24,25].

### 4.3. Injury Severity (TBSA) and Anatomical Distribution

Reporting of depth-stratified severity remains uncommon in the global epidemiological burn literature. Several systematic reviews have noted that most epidemiological studies report TBSA but not standardised depth categories, limiting cross-cohort comparability of injury severity [6,13,16]. Most studies focused solely on burn size and did not account for burn depth [13]. Across high-quality reviews, most admitted cases cluster below 20% TBSA; the MENA pooled mean BSAB is 17.2%, with second-degree burns comprising 56.5% [16]. Global evidence indicates similar distributions, with extremities frequently involved; the MENA meta-analysis reports upper-limb involvement at 51.7%, aligning with international patterns [13,16].

The TBSA involvement at JUH (median 7%, IQR 5.32) falls within established global parameters for hospital-admitted burn patients. An 8-year retrospective study from China, including 9779 burn patients, found that 95.54% had TBSA <50%, with most cases involving smaller surface areas [15]. A recent study at the Tertiary Indian Burn Care Centre found that the median total body surface area (TBSA) burned was 20% [14]. The JUH findings of 73.9% partial-thickness burns, 13.0% full-thickness burns, and 2.1% superficial burns align with international patterns, in which partial-thickness burns predominate among hospital-admitted patients [15].

In 1980 at JUH, limb involvement predominated, particularly the upper limbs in fire burns and the trunk in scalds. Our data corroborate this general pattern, with multiple sites and lower limbs most frequently affected (32.0% and 19.8%, respectively) [10]. However, classification of burn depth and TBSA is now more precise in our cohort: partial-thickness burns accounted for 73.9% of cases, with a median TBSA of 7%. Such standardised reporting, absent in the earlier study by Muhtaseb et al., underscores advances in clinical documentation and burn-care stratification at JUH.

A study in China from 2009 to 2018, involving a total of 333,995 burn inpatients, found, similar to our study, that most commonly, burns affected multiple burn sites (230,090, 68.89% [15]). Most informatively, the WHO Global Burn Registry paediatric analysis and a 2023 systematic review and meta-analysis of paediatric emergency department burn cohorts, pooling 828,538 children from 22 studies, consistently identify the extremities and multi-site involvement as the dominant anatomical patterns in paediatric burn populations [4,27].

However, this finding aligns with recent large-scale temporal analyses that found no predictable annual pattern in burn admissions [28].

The WHO Global Burn Registry (GBR) paediatric analysis by Jordan et al. (3649 children from 20 predominantly middle-income countries) documented that 52% of admitted paediatric patients sustained major burns (≥15% TBSA), that 48% underwent surgery during the index hospitalisation, and that critical-care capacity was reported as “limited” for 23% of paediatric admissions [4].

### 4.4. Treatment Modalities and Severity Correlations

International reviews emphasise the diffusion of early excision/grafting, infection control, and multidisciplinary rehabilitation as key advances associated with improved outcomes and shorter LOS, conditional on TBSA and inhalation status [23].

The treatment patterns at JUH demonstrated significant associations between burn severity and intervention requirements, consistent with global standards of care. The finding that full-thickness burns more frequently required surgical grafting while partial-thickness burns were managed conservatively aligns with established international protocols [26,29,30,31]. The relationship between electrical burns and higher surgical intervention rates corresponds to global patterns where electrical injuries are associated with deeper tissue damage requiring more aggressive surgical management [26,29,30,31]. This confirms that the JUH patterns align with global surgical management principles documented in international burn care literature [30].

Al-Muhtasib et al. reported limited surgical interventions, only two amputations and two craniotomies, with irregular follow-up and many discharges against medical advice [10]. The current study demonstrates a more complex therapeutic landscape, including structured surgical management (e.g., grafting, escharotomy) and detailed outcome monitoring. Regression analysis identified grafting, adverse clinical events, and larger TBSA as independent predictors of prolonged hospitalisation, reflecting analytical sophistication and a capacity for multidisciplinary care unavailable four decades ago.

### 4.5. Predictors of Hospital Length of Stay

Multivariable analyses consistently identify TBSA, burn depth, inhalation injury, and infection as independent LOS drivers. An Egyptian cohort found infection, depth, TBSA%, and inhalation injury to be most influential for LOS prediction. Mean LOS was identified as 24.2 days and mortality at 9.8% [32]. Competing-risk analyses of >95,000 patients admitted with an acute burn injury to 80 tertiary American Burn Association burn centres from 2000 to 2009 showed TBSA, age, and inhalation injury significantly impact the subdistribution hazard for discharge (all *p* < 0.001), with burn size governing early outcomes and age increasingly influential later in admission [33]. Paediatric LOS determinants in Australia similarly include %TBSA and full-thickness injury, with extended stays linked to remoteness and flame burns [34]. Beyond biological severity, large-database modelling from England indicates that socioeconomic deprivation and mental health comorbidity segments are associated with longer LOS and attenuate the relative impact of severity variables in prediction models [35].

The JUH regression model, which explained 29.9% of the variance in length of stay, identified several predictors consistent with the global burn-outcome literature. The association between surgical grafting and a prolonged stay (+14.6 days) aligns with international patterns in which surgical interventions extend hospitalisation [36,37]. The finding that TBSA was a strong predictor of prolonged stay (+32.1 days per unit increase) corresponds to established global predictors of burn outcomes [36,37,38].

The association between burn degree and hospital stay, with full-thickness burns requiring longer hospitalisations, aligns with international patterns consistently reported in the burn literature. Studies from multiple regions have identified TBSA and burn depth as primary determinants of hospital length of stay [36,37,38].

The identification of adverse clinical outcomes as significant predictors of prolonged hospitalisation (mortality, atrial fibrillation, aphasia, end-stage kidney disease, and reactive thrombocytosis) reflects the complex medical management requirements for severely burned patients globally. These findings align with international studies identifying complications as major drivers of extended hospitalisation and poor outcomes [13,39].

### 4.6. Mortality, LOS, and Regional Benchmarks

Mortality has decreased markedly from 11.8% (40/338) in 1980 to 2.1% (12/575) in the present cohort. However, the main causes of death have remained the same, indicating persistent systemic complications among severe cases [10]. The study from northern Jordan documented flame burns accounting for 60% of cases, and scald burns for 23%, representing a different aetiology distribution compared to JUH, possibly reflecting differences in study populations or referral patterns [17,23]. A recent study on the predictors of mortality in the ICU in JUH found infections to be a significant predictor of mortality, [40], which, along with other factors, leads us to attribute the comparatively low JUH mortality to case-mix factors, predominantly low-TBSA scald burns in children, rather than superior care infrastructure, and should be interpreted accordingly. Further studies are needed to conclude an acausal relationship.

Global syntheses report concurrent declines in incidence, severity, LOS, and mortality in very-high-development settings, with more heterogeneous patterns elsewhere [13]. The MENA meta-analysis yields a pooled LOS of 11.2 days and a mortality of 9.1%, values broadly overlapping with historical international estimates when matched on TBSA and case-mix [16].

### 4.7. Burn Wound Infection and Complications

Although systematic infection surveillance data were not captured in this dataset, sepsis was the second leading cause of in-hospital death (n = 3, 25% of fatal cases), and infection-related complications emerged as independent drivers of prolonged LOS in the regression model. This aligns with global evidence identifying burn wound infection—particularly with multi-drug-resistant gram-negative organisms, such as Pseudomonas aeruginosa, Klebsiella pneumoniae, and Acinetobacter baumannii—as a principal determinant of morbidity and mortality in hospitalised burn patients [41,42,43,44]. In low- and middle-income settings comparable to Jordan, reported burn wound infection rates range from 25% to over 60%, substantially exceeding high-income burn centre benchmarks, largely due to differences in wound care infrastructure, isolation capacity, and antimicrobial stewardship [41,42,43,44]. The absence of systematic infection data from this cohort represents a critical evidence gap; future prospective burn registries at JUH should include standardised wound-culture protocols, organism identification, and antibiogram results. In the interim, early surgical debridement, protocolised dressing regimens, and antimicrobial stewardship programmes aligned with regional resistance patterns should be prioritised as quality-improvement targets.

## 5. Limitations and Future Directions

Our study has several limitations that should be acknowledged. First, its retrospective design inherently limits causal inference and is subject to incomplete or inaccurate documentation in medical records. Despite careful data extraction and cleaning, missing information was unavoidable, particularly regarding burn depth and degree in some cases, which may have influenced the precision of certain statistical analyses.

Second, although the total sample size was relatively large (n = 575), the number of deceased patients was small (n = 12). This limited our ability to conduct a robust multivariate model to identify predictors of mortality. Consequently, mortality-related findings should be interpreted descriptively rather than inferentially.

Third, this was a single-centre study conducted at Jordan University Hospital, a tertiary referral centre in Amman that benefits from better infrastructure, specialised burn units, and greater resource availability than hospitals in other regions of Jordan. Therefore, the findings may not fully represent national patterns or outcomes in peripheral or rural healthcare settings.

Fourth, the 1976–1980 JUH cohort reported by El-Muhtaseb et al. (1983) [10] was assembled from contemporaneous paper-based clinical records, whereas the present 2016–2020 cohort was identified through ICD-10-coded queries of the hospital information system, cross-referenced with burn-unit, emergency department, operating room, and ICU records (Section 2.3). These differences in case-ascertainment infrastructure are real, cannot be reconciled retrospectively, and may bias inter-cohort comparisons in either direction—toward over-ascertainment in the contemporary cohort (through multi-source identification) or toward under-ascertainment in the historical cohort (through reliance on a narrower paper-based record set). Accordingly, the comparative figures presented should be read as a structured institution-level time series rather than as a controlled patient-level comparison. We believe their value lies in informing institutional and regional health services planning over a 40-year horizon, not in establishing causal inferences about temporal change.

We further acknowledge that the decision to retain “unknown” as an explicit category for burn depth and burn mechanism—adopted to preserve sample size and analytical power—introduces a missingness-as-category bias. The “unknown” group may differ systematically from the categorised groups on unmeasured clinical features, in a direction the model cannot determine. The negative LOS coefficient observed for “unknown depth” in Table 3 is most plausibly explained not by an intrinsic effect of depth uncertainty but by selective non-recording of severity in clinically milder, briefer admissions, in which detailed depth charting was likely deemed unnecessary.

Additionally, some variables, such as socioeconomic status, education level, and preventive measures at the time of injury, were not systematically recorded and thus could not be analysed. These unmeasured confounders may have influenced the observed associations between demographic or etiologic factors and clinical outcomes.

Finally, although the study used standardised definitions for burn degree and total body surface area, inter-observer variability in clinical assessment and documentation cannot be excluded, particularly given the multiclinician charting typical of retrospective designs.

## 6. Conclusions

The epidemiological patterns at JUH demonstrate both global convergence and regional specificities that reflect local risk factors, healthcare delivery systems, and population characteristics. While the predominance of paediatric scald injuries and home-based accidents aligns with global patterns, the specific aetiology, distributions and outcome predictors require context-specific prevention and treatment strategies. Future research should focus on establishing regional burn registries to enable more robust epidemiological comparisons and evidence-based prevention strategies tailored to the characteristics of the MENA region.

In summary, our findings (i) provide a detailed contemporary clinical and demographic profile of acute burn admissions at Jordan University Hospital across 2016–2020, (ii) demonstrate a marked reduction in in-hospital mortality compared with the 1976–1980 JUH cohort (2.1% vs. 11.8%), and (iii) identify total body surface area, burn depth, surgical grafting, and in-hospital adverse events as the principal independent predictors of hospital length of stay. The persistence of domestic scald injuries—particularly among children—highlights the continuing need for targeted household and child-safety education; the emergence of electrical and chemical burns suggests evolving occupational and household hazards that warrant further study. We do not, however, claim from this descriptive single-centre design to have demonstrated improvements in prevention or emergency response per se; such claims would require dedicated outcome-evaluation studies. The near-equal gender representation, the unchanged predominance of home-based injury contexts, and the marked reduction in mortality together indicate areas where public health interventions and continued investment in specialised burn care can be productively focused.

## Figures and Tables

**Table 1:** Demographic and clinical characteristics of patients by burn degree (N = 575).

Variable	Category	Superficial n (%)	Partial Thickness n (%)	Full Thickness n (%)	Unknown n (%)	Total (n)	*p*-Value
**Sex**	Male	3 (1.0)	211 (73.0)	42 (14.5)	33 (11.4)	289	0.238
	Female	9 (3.1)	214 (74.8)	33 (11.5)	30 (10.5)	286	
**Age**	Median (IQR)	37.5 (20)	13 (36)	27 (33)	23 (34)	19 (37)	<0.001
**City of Residence**	Ajloun	0 (0.0)	1 (50.0)	0 (0.0)	1 (50.0)	2	0.164
	Al-Karak	1(9.1)	4 (36.4)	4 (36.4)	2 (18.2)	11	
	Al-Mafraq	0 (0.0)	3 (100.0)	0 (0.0)	0 (0.0)	3	
	Al-Tafilah	0 (0.0)	2 (66.7)	1(33.3)	0 (0.0)	3	
	Al-Zarqa’a	0 (0.0)	31 (72.1)	6 (14.0)	6 (14.0)	43	
	Amman	10 (2.9)	267 (76.9)	38 (11.0)	32 (9.2)	347	
	Aqaba	0 (0.0)	4 (100.0)	0 (0.0)	0 (0.0)	4	
	Balqa	1 (0.8)	82 (68.3)	17 (14.2)	20 (16.7)	120	
	Irbid	0 (0.0%)	6 (85.7)	0 (0.0)	1 (14.3)	7	
	Jerash	0 (0.0%)	12 (75.0)	3 (18.8)	1 (6.2)	16	
	Ma’an	0 (0.0%)	3 (60.0)	2 (40.0)	0 (0.0%)	5	
	Madaba	0 (0.0%)	10 (71.4)	4 (28.6)	0 (0.0%)	14	
**Age group (2-cat)**	Child	2 (0.7)	231 (80.5)	27 (9.4)	27 (9.4)	287	0.001
	Adult	10 (3.5)	194 (67.4)	48 (16.7)	36 (12.5)	288	
**Nationality**	Jordanian	12 (2.2)	402 (73.8)	71 (13.0)	60 (11.0)	545	>0.999
	Non-Jordanian	0 (0.0)	23 (76.7)	4 (13.3)	3 (10.0)	30	
**Occupation**	Freelancer	1 (2.3)	24 (55.8)	11 (25.6)	7 (16.3)	43	<0.001
	Gov. employee	8 (6.2)	84 (65.6)	22 (17.2)	14 (10.9)	128	
	Housewife	0 (0.0)	16 (80.0)	4 (20.0)	0 (0.0)	20	
	Preschooler	0 (0.0)	100 (88.5)	10 (8.8)	3 (2.7)	113	
	Private sector	0 (0.0)	3 (100.0)	0 (0.0)	0 (0.0)	3	
	Retired	1 (1.7)	44 (74.6)	6 (10.2)	8 (13.6)	59	
	Student	2 (1.0)	154 (73.7)	22 (10.5)	31 (14.8)	209	
**Season**	Autumn	1 (0.8)	104 (81.3)	14 (10.9)	9 (7.0)	128	0.258
	Spring	3 (2.2)	91 (66.9)	21 (15.4)	21 (15.4)	136	
	Summer	3 (1.8)	122 (74.8)	18 (11.0)	20 (12.3)	163	
	Winter	5 (3.4)	108 (73.0)	22 (14.9)	13 (8.8)	148	
**Comorbidities**	None	9 (1.8)	369 (73.4)	67 (13.3)	58 (11.5)	503	0.345
	Any	3 (4.2)	56 (77.8)	8 (11.1)	5 (6.9)	72	
**Cause of burn**	Scald	1 (0.3)	298 (85.4)	34 (9.7)	16 (4.6)	349	<0.001
	Flame	6 (5.4)	78 (69.6)	15 (13.4)	13 (11.6)	112	
	Electrical	0 (0.0)	2 (7.1)	9 (32.1)	17 (60.7)	28	
	Chemical	3 (11.5)	18 (69.2)	2 (7.7)	3 (11.5)	26	
	Contact	0 (0.0)	18 (72.0)	5 (20.0)	2 (8.0)	25	
	Other	2 (11.8)	8 (47.1)	4 (23.5)	3 (17.6)	17	
	Unknown	0 (0.0)	3 (16.7)	6 (33.3)	9 (50.0)	18	
**Site of burn**	Multiple	6 (3.3)	149 (81.0)	22 (12.0)	7 (3.8)	184	0.001
	Head/Neck	2 (5.3)	28 (73.7)	4 (10.5)	4 (10.5)	38	
	Upper limb	3 (3.2)	66 (71.0)	9 (9.7)	15 (16.1)	93	
	Lower limb	0 (0.0)	90 (78.9)	18 (15.8)	6 (5.3)	114	
	Trunk	0 (0.0)	45 (91.8)	1 (2.0)	3 (6.1)	49	
	Genital/Perineum	0 (0.0)	2 (66.7)	0 (0.0)	1 (33.3)	3	
	Unknown	1 (1.1)	45 (47.9)	21 (22.3)	27 (28.7)	94	
**Place of Accident**	Home (Unspecified)	12 (2.3)	395 (74.2)	67 12.6)	58 (10.9)	532	0.424
	Hospital	0 (0.0)	1 (100.0)	0 (0.0)	0 (0.0)	1	
	Kitchen	0 (0.0)	11 (78.6)	2 (14.3)	1 (7.1)	14	
	Road Traffic Accident	0 (0.0)	1 (50.0)	1 (50.0)	0 (0.0)	2	
	School	0 (0.0)	0 (0.0)	0 (0.0)	1 (100.0)	1	
	Shower	0 (0.0)	2 (100.0)	0 (0.0)	0 (0.0)	2	
	Street	0 (0.0)	0	1 (50.0)	1 (50.0)	2	
	Swimming Pool	0 (0.0)	1 (100.0)	0 (0.0)	0 (0.0)	1	
	Work	0 (0.0)	14 (70.0)	4 (20.0)	2 (10.0)	20	
**Treatment modality**	Conservative	10 (2.6)	311 (81.4)	27 (7.1)	34 (8.9)	382	<0.001
	Surgical grafting	2 (1.3)	109 (68.1)	42 (26.3)	7 (4.4)	160	
	Other	0 (0.0)	2 (20.0)	2 (20.0)	6 (60.0)	10	
	Discharged untreated	0 (0.0)	2 (40.0)	1 (20.0)	2 (40.0)	5	
	Unknown	0 (0.0)	1 (5.6)	3 (16.7)	14 (77.8)	18	
**Duration of stay**	Median (IQR)	3 (5)	6(9)	12(28)	3 (6)	6(10)	<0.001
**TBSA%**	Median (IQR)	9.32% (0.00%)	6.00% (5.32%)	9.32% (14.00%)	9.32% (0.00%)	7% (5.32%)	<0.001

**Table 2:** Demographic and clinical characteristics of patients by type of burn (N = 575).

Variable	Category	Scald n (%)	Flame n (%)	Electrical n (%)	Chemical n (%)	Contact n (%)	Other n (%)	Unknown n (%)	*p*-Value
**Sex**	Male	159 (55.0)	63 (21.8)	22 (7.6)	17 (5.9)	11 (3.8)	8 (2.8)	9 (3.1)	0.011
	Female	190 (66.4)	49 (17.1)	6 (2.1)	9 (3.1)	14 (4.9)	9 (3.1)	9 (3.1)	
**Age**	Median (IQR)	7 (33)	34.5 (27)	23 (27)	33 (21)	7 (22)	37 (48)	34 (42)	<0.001
**City of Residence**	Ajloun	3 (60.0)	1 (20.0)	0 (0.0)	0 (0.0)	0 (0.0)	0 (0.0)	1 (20.0)	0.105
	Al-Karak	15 (65.2)	4 (17.4)	0 (0.0)	1 (4.3)	2 (8.7)	0 (0.0)	1 (4.3)	
	Al-Mafraq	6 (46.2)	2 (15.4)	1 (7.7)	1 (7.7)	1 (7.7)	1 (7.7)	1 (7.7)	
	Al-Tafilah	2 (66.7)	0 (0.0)	0 (0.0)	1 (33.3)	0 (0.0)	0 (0.0)	0 (0.0)	
	Al-Zarqa’a	22 (62.9)	6 (17.1)	2 (5.7)	2 (5.7)	2 (5.7)	1 (2.9)	0 (0.0)	
	Amman	210 (59.5)	72 (20.4)	19 (5.4)	19 (5.4)	13 (3.7)	9 (2.5)	11 (3.1)	
	Aqaba	7 (58.3)	4 (33.3)	0 (0.0)	1 (8.3)	0 (0.0)	0 (0.0)	0 (0.0)	
	Balqa	12 (70.6)	2 (11.8)	0 (0.0)	1 (5.9)	0 (0.0)	1 (5.9)	1 (5.9)	
	Irbid	51 (68.9)	13 (17.6)	2 (2.7)	3 (4.1)	1 (1.4)	2 (2.7)	2 (2.7)	
	Jerash	6 (85.7)	1 (14.3)	0 (0.0)	0 (0.0)	0 (0.0)	0 (0.0)	0 (0.0)	
	Ma’an	2 (40.0)	1 (20.0)	1 (20.0)	0 (0.0)	0 (0.0)	0 (0.0)	1 (20.0)	
	Madaba	3 (50.0)	2 (33.3)	0 (0.0)	1 (16.7)	0 (0.0)	0 (0.0)	0 (0.0)	
**Age group (2-cat)**	Child	218 (76.0)	23 (8.0)	13 (4.5)	6 (2.1)	16 (5.6)	6 (2.1)	5 (1.7)	<0.001
	Adult	131 (45.5)	89 (30.9)	15 (5.2)	20 (6.9)	9 (3.1)	11 (3.8)	13 (4.5)	
**Nationality**	Jordanian	337 (61.8)	99 (18.2)	28 (5.1)	26 (4.8)	24 (4.4)	15 (2.8)	16 (2.9)	0.009
	Non-Jordanian	12 (40.0)	13 (43.3)	0 (0.0)	0 (0.0)	1 (3.3)	2 (6.7)	2 (6.7)	
**Occupation**	Freelancer	13 (30.2)	16 (37.2)	4 (9.3)	3 (7.0)	1 (2.3)	2 (4.7)	4 (9.3)	<0.001
	Gov. employee	58 (45.3)	43 (33.6)	9 (7.0)	9 (7.0)	1 (0.8)	3 (2.3)	5 (3.9)	
	Housewife	11 (55.0)	8 (40.0)	0 (0.0)	0 (0.0)	1 (5.0)	0 (0.0)	0 (0.0)	
	Preschooler	96 (85.0)	2 (1.8)	2 (1.8)	4 (3.5)	7 (6.2)	1 (0.9)	1 (0.9)	
	Private sector	1 (33.3)	0 (0.0)	0 (0.0)	1 (33.3)	1 (33.3)	0 (0.0)	0 (0.0)	
	Retired	34 (57.6)	9 (15.3)	1 (1.7)	5 (8.5)	3 (5.1)	5 (8.5)	2 (3.4)	
	Student	136 (65.1)	34 (16.3)	12 (5.7)	4 (1.9)	11 (5.3)	6 (2.9)	6 (2.9)	
**Season**	Autumn	82 (64.1)	24 (18.8)	6 (4.7)	7 (5.5)	4 (3.1)	3 (2.3)	2 (1.6)	0.921
	Spring	77 (56.6)	25 (18.4)	8 (5.9)	6 (4.4)	11 (8.1)	4 (2.9)	5 (3.7)	
	Summer	99 (60.7)	31 (19.0)	9 (5.5)	8 (4.9)	4 (2.5)	6 (3.7)	6 (3.7)	
	Winter	91 (61.5)	32 (21.6)	5 (3.4)	5 (3.4)	6 (4.1)	4 (2.7)	5 (3.4)	
**Comorbidities**	None	314 (62.4)	89 (17.7)	27 (5.4)	24 (4.8)	22 (4.4)	11 (2.2)	16 (3.2)	0.006
	Any	35 (48.6)	23 (31.9)	1 (1.4)	2 (2.8)	3 (4.2)	6 (8.3)	2 (2.8)	
**Degree of burn**	Superficial	1 (8.3)	6 (50.0)	0 (0.0)	3 (25.0)	0 (0.0)	2 (16.7)	0 (0.0)	<0.001
	Partial thickness	298 (70.1)	78 (18.4)	2 (0.5)	18 (4.2)	18 (4.2)	8 (1.9)	3 (0.7)	
	Full thickness	34 (45.3)	15 (20.0)	9 (12.0)	2 (2.7)	5 (6.7)	4 (5.3)	6 (8.0)	
	Unknown	16 (25.4)	13 (20.6)	17 (27.0)	3 (4.8)	2 (3.2)	3 (4.8)	9 (14.3)	
**Site of burn**	Multiple	107 (58.2)	58 (31.5)	1 (0.5)	4 (2.2)	4 (2.2)	5 (2.7)	5 (2.7)	<0.001
	Head/Neck	12 (31.6)	16 (42.1)	2 (5.3)	3 (7.9)	5 (13.2)	0 (0.0)	0 (0.0)	
	Upper limb	55 (59.1)	10 (10.8)	13 (14.0)	6 (6.5)	9 (9.7)	0 (0.0)	0 (0.0)	
	Lower limb	84 (73.7)	12 (10.5)	1 (0.9)	4 (3.5)	7 (6.1)	5 (4.4)	1 (0.9)	
	Trunk	40 (81.6)	3 (6.1)	0 (0.0)	4 (8.2)	0 (0.0)	1 (2.0)	1 (2.0)	
	Genital/Perineum	2 (66.7)	0 (0.0)	0 (0.0)	1 (33.3)	0 (0.0)	0 (0.0)	0 (0.0)	
	Unknown	49 (52.1)	13 (13.8)	11 (11.7)	4 (4.3)	0 (0.0)	6 (6.4)	11 (11.7)	
**Place of Accident**	Home (Unspecified)	340 (63.9)	95 (17.9)	22 (4.1)	24 (4.5)	21 (3.9)	12 (2.3)	18 (3.4)	<0.001
	Hospital	2 (40.0)	1 (20.0)	0 (0.0)	0 (0.0)	0 (0.0)	1 (20.0)	1 (20.0)	
	Kitchen	5 (35.7)	5 (35.7)	1 (7.1)	0 (0.0)	3 (21.4)	0 (0.0)	0 (0.0)	
	Road Traffic Accident	0 (0.0)	0 (0.0)	1 (100.0)	0 (0.0)	0 (0.0)	0 (0.0)	0 (0.0)	
	School	1 (50.0)	0 (0.0)	0 (0.0)	0 (0.0)	1 (50.0)	0 (0.0)	0 (0.0)	
	Shower	1 (33.3)	1 (33.3)	0 (0.0)	0 (0.0)	1 (33.3)	0 (0.0)	0 (0.0)	
	Street	2 (40.0)	2 (40.0)	0 (0.0)	1 (20.0)	0 (0.0)	0 (0.0)	0 (0.0)	
	Swimming Pool	1 (100.0)	0 (0.0)	0 (0.0)	0 (0.0)	0 (0.0)	0 (0.0)	0 (0.0)	
	Work	20 (50.0)	11 (27.5)	5 (12.5)	1 (2.5)	1 (2.5)	1 (2.5)	1 (2.5)	
**Treatment modality**	Conservative	246 (64.4)	73 (19.1)	18 (4.7)	13 (3.4)	15 (3.9)	10 (2.6)	7 (1.8)	<0.001
	Surgical grafting	91 (56.9)	32 (20.0)	4 (2.5)	13 (8.1)	9 (5.6)	7 (4.4)	4 (2.5)	
	Other	4 (40.0)	2 (20.0)	2 (20.0)	0 (0.0)	0 (0.0)	0 (0.0)	2 (20.0)	
	Discharged untreated	2 (40.0)	1 (20.0)	1 (20.0)	0 (0.0)	0 (0.0)	0 (0.0)	1 (20.0)	
	Unknown	6 (33.3)	4 (22.2)	3 (16.7)	0 (0.0)	1 (5.6)	0 (0.0)	4 (22.2)	
**Duration of stay**	Median (IQR)	6 (8)	6.5 (14)	1.5 (5)	2.5 (5)	4 (8)	4 (15)	9 (22)	<0.001
**TBSA%**	Median (IQR)	6.00% (5.32%)	9.00% (10.00%)	9.32% (0.00%)	4.75% (8.32%)	4.00% (4.00%)	9.00% (3.82%)	13.00% (7.18%)	<0.001

**Table 3:** Multiple linear regression model to predict the length of stay.

			95% Confidence Interval			
Predictor	Estimate	SE	Lower	Upper	t	*p*
Intercept ᵃ	7.1979	3.57	0.177	14.219	2.0138	0.045
Surgical Grafting:						
Yes—No	14.5518	3.62	7.434	21.670	4.0156	<0.001
Escharectomy Treatment:						
Yes—No	−11.4919	3.66	−18.674	−4.310	−3.1430	0.002
Deceased:						
Yes—No	16.1682	4.68	6.970	25.366	3.4529	<0.001
Atrial Fibrillation:						
Yes—No	44.0673	8.02	28.323	59.811	5.4980	<0.001
Aphasic:						
Yes—No	58.8142	14.23	30.870	86.758	4.1343	<0.001
End Stage Kidney Disease on dialysis:						
Yes—No	53.4858	13.74	26.502	80.469	3.8936	<0.001
Reactive Thrombocytosis:						
Yes—No	35.8825	13.95	8.478	63.287	2.5720	0.010
Address:						
Ajloun—Amman	2.2055	9.84	−17.126	21.537	0.2241	0.823
Al-Karak—Amman	−2.1872	4.42	−10.872	6.497	−0.4947	0.621
Al-Mafraq—Amman	−4.2486	7.96	−19.892	11.395	−0.5335	0.594
Al-Tafilah—Amman	−11.2089	8.12	−27.150	4.732	−1.3812	0.168
Al-Zarqa’a—Amman	5.0409	2.22	0.675	9.407	2.2678	0.024
Aqaba—Amman	2.7440	6.90	−10.814	16.302	0.3976	0.691
Balqa—Amman	0.6781	1.48	−2.223	3.579	0.4591	0.646
Irbid—Amman	9.9009	5.35	−0.618	20.419	1.8490	0.065
Jerash—Amman	4.4553	3.52	−2.460	11.370	1.2656	0.206
Ma’an—Amman	−6.0540	6.35	−18.526	6.418	−0.9535	0.341
Madaba—Amman	−3.8605	3.80	−11.321	3.600	−1.0164	0.310
Percentage of burn	32.0647	6.90	18.509	45.621	4.6463	<0.001
Degree of Burn:						
Partial thickness (2nd degree)—Full thickness (3rd degree)	−8.7160	1.98	−12.596	−4.836	−4.4128	<0.001
Superficial (1st degree)—Full thickness (3rd degree)	−9.4597	4.51	−18.317	−0.602	−2.0979	0.036
Unknown—Full thickness (3rd degree)	−8.9430	2.56	−13.978	−3.908	−3.4892	<0.001
Cause of Burn:						
Contact burn—Chemical burn	5.8845	3.95	−1.881	13.650	1.4884	0.137
Electrical burn—Chemical burn	−0.0557	4.09	−8.094	7.983	−0.0136	0.989
Flame burn—Chemical burn	7.4831	3.12	1.356	13.611	2.3989	0.017
Other/Unknown—Chemical burn	0.6834	4.49	−8.132	9.499	0.1523	0.879
Scald burn—Chemical burn	6.0717	2.94	0.293	11.850	2.0640	0.039
Unknown—Chemical burn	2.7597	4.69	−6.447	11.967	0.5888	0.556

ᵃ Represents reference level.

## Data Availability

The original contributions presented in the study are included in the article, further inquiries can be directed to the corresponding author.

## References

[1] World Health Organization Burns. Fact Sheet. https://www.who.int/news-room/fact-sheets/detail/burns.

[2] Yakupu A., Zhang J., Dong W., Song F., Dong J., Lu S. (2022). The Epidemiological Characteristic and Trends of Burns Globally. BMC Public Health.

[3] Peck M.D. (2011). Epidemiology of Burns throughout the World. Part I: Distribution and Risk Factors. Burns.

[4] Jordan K.C., Di Gennaro J.L., von Saint André-von Arnim A., Stewart B.T. (2022). Global Trends in Pediatric Burn Injuries and Care Capacity from the World Health Organization Global Burn Registry. Front. Pediatr..

[5] Mehta K., Arega H., Smith N.L., Li K., Gause E., Lee J., Stewart B. (2022). Gender-Based Disparities in Burn Injuries, Care and Outcomes: A World Health Organization (WHO) Global Burn Registry Cohort Study. Am. J. Surg..

[6] Othman N., Kendrick D. (2010). Epidemiology of Burn Injuries in the East Mediterranean Region: A Systematic Review. BMC Public Health.

[7] Sharma P.N., Bang R.L., Al-Fadhli A.N., Sharma P., Bang S., Ghoneim I.E. (2006). Paediatric Burns in Kuwait: Incidence, Causes and Mortality. Burns.

[8] Groohi B., Alaghehbandan R., Lari A.R. (2002). Analysis of 1089 Burn Patients in Province of Kurdistan, Iran. Burns.

[9] Al-Shehri M. (2004). The Pattern of Paediatric Burn Injuries in Southwestern, Saudi Arabia. West Afr. J. Med..

[10] El-Muhtaseb H., Qaryoute S., Ragheb S.A. (1983). Burn Injuries in Jordan: A Study of 338 Cases. Burns.

[11] Hamdan F.R. (2018). Epidemiology and Management Outcome of Burn Injury in Jordanian Hospitals. Int. J. Nurs..

[12] Najafali D., Rezaei S., Liu H., Arellano J., Reiche E., Tran Q., Patel S., Nthumba P., Stams V., Egro F. (2025). 953 Global Disparities in Burns: Analyzing Outcomes from the WHO Global Burn Registry Across Resource Settings. J. Burn Care Res..

[13] Smolle C., Cambiaso-Daniel J., Forbes A.A., Wurzer P., Hundeshagen G., Branski L.K., Huss F., Kamolz L.P. (2017). Recent Trends in Burn Epidemiology Worldwide: A Systematic Review. Burns.

[14] Kumar N., Eisner Z.J., Saha S., Kumar V., Singhal M. (2025). Insight on Pediatric Burn Morbidity and Mortality at a Tertiary Indian Burn Care Center: A Case for Burn Prevention. J. Burn Care Res..

[15] Zheng Y., Lin G., Zhan R., Qian W., Yan T., Sun L., Luo G. (2019). Epidemiological Analysis of 9,779 Burn Patients in China: An Eight-Year Retrospective Study at a Major Burn Center in Southwest China. Exp. Ther. Med..

[16] Elshahidi M.H. (2024). Clinico-Demographic Profile of Burns in the Middle-East and North-Africa (MENA) Region: A Systematic Review and Meta-Analysis. Discov. Public Health.

[17] Bataineh Z.A., Al Quran T.M., Al Balas H., Khammash M.R. (2018). Pattern of Burn Injury at North of Jordan. Int. J. Burn. Trauma.

[18] Almarghoub M.A., Alotaibi A.S., Alyamani A., Alfaqeeh F.A., Almehaid F.F., Al-Qattan M.M., Kattan A.E. (2020). The Epidemiology of Burn Injuries in Saudi Arabia: A Systematic Review. J. Burn Care Res..

[19] Khashaba H.A., Al-Fadhli A.N., Al-Tarrah K.S., Wilson Y.T., Moiemen N. (2012). Epidemiology and Outcome of Burns at the Saud Al Babtain Burns, Plastic Surgery and Reconstructive Center, Kuwait: Our Experience over Five Years (from 2006 to 2010). Ann. Burn. Fire Disasters.

[20] Rayner R., Prentice J. (2011). Paediatric Burns: A Brief Global Review. Wound Pract. Res..

[21] Peden M., Oyegbite K., Ozanne-Smith J., Hyder A.A., Branche C., Rahman A.K.M.F., Rivara F., Bartolomeos K. (2008). World Report on Child Injury Prevention.

[22] Department of Statistics [Jordan], ICF (2024). Jordan Population and Family Health Survey 2023.

[23] Jeschke M.G., Van Baar M.E., Choudhry M.A., Chung K.K., Gibran N.S., Logsetty S. (2020). Burn Injury. Nat. Rev. Dis. Prim..

[24] Setiawan D., Haryono W. (2020). Epidemiology and Characteristics of Burn Patients in Dr. Soedarso General Hospital during 2017–2020: Retrospective Study. Open Access Maced. J. Med. Sci..

[25] Forbinake N.A., Ohandza C.S., Fai K.N., Agbor V.N., Asonglefac B.K., Aroke D., Beyiha G. (2020). Mortality Analysis of Burns in a Developing Country: A CAMEROONIAN Experience. BMC Public Health.

[26] Jain S., Singla C., Toor S., Bhatti D.J., Gupta P. (2023). Epidemiology and Acute Management of High Tension Electrical Burns in a Rural-Based Medical College. Ambul. Surg..

[27] Nassar J.Y., Al Qurashi A.A., Albalawi I.A., Nukaly H.Y., Halawani I.R., Abumelha A.F., Al Dwehji A.M.O., Alhartani M.M., Asaad A., Alnajashi A. (2023). Pediatric Burns: A Systematic Review and Meta-Analysis on Epidemiology, Gender Distribution, Risk Factors, Management, and Outcomes in Emergency Departments. Cureus.

[28] Beyene R.T., Stonko D.P., Gondek S.P., Morrison J.J., Dennis B.M. (2023). Identifying Temporal Variations in Burn Admissions. PLoS ONE.

[29] McCann C., Watson A., Barnes D. (2022). Major Burns: Part 1. Epidemiology, Pathophysiology and Initial Management. BJA Educ..

[30] Paredes K.A., Rojas J.C.S., Valdés J.R.F., Castillo J.L., Quevedo M.M., Delgado F.J.M., de la Cruz Durán H.A., Mendoza C.L.N., Vazquez E.J.N., Paredes K.A. (2024). A Comparative Analysis of the Outcomes of Various Graft Types in Burn Reconstruction Over the Past 24 Years: A Systematic Review. Cureus.

[31] Friedstat J., Brown D.A., Levi B. (2017). Chemical, Electrical, and Radiation Injuries. Clin. Plast. Surg..

[32] AbdelWahab M.E., Sadaka M.S., Elbana E.A., Hendy A.A. (2018). Evaluation of Prognostic Factors Affecting Lenght of Stay in Hospital and Mortality Rates in Acute Burn Patients. Ann. Burn. Fire Disasters.

[33] Taylor S.L., Sen S., Greenhalgh D.G., Lawless M., Curri T., Palmieri T.L. (2015). A Competing Risk Analysis for Hospital Length of Stay in Patients with Burns. JAMA Surg..

[34] Ryder C., Mackean T., Hunter K., Towers K., Rogers K., Holland A.J.A., Ivers R. (2020). Factors Contributing to Longer Length of Stay in Aboriginal and Torres Strait Islander Children Hospitalised for Burn Injury. Inj. Epidemiol..

[35] Onah C.N., Allmendinger R., Handl J., Dunn K.W. (2021). Surviving Burn Injury: Drivers of Length of Hospital Stay. Int. J. Environ. Res. Public Health.

[36] Zhu X.M., Tedesco D., Gallo L., Shahrokhi S., Jeschke M.G. (2024). 126 Predictors of Lengthened Admission in Adult Burn Patients, a Secondary Analysis of 1796 Cases. J. Burn Care Res..

[37] Shah P., Bhavsar D., Nazir N., Reynolds J., Vazquez Machado M.C., Work E.D. (2023). 285 Determinants of Mortality Amongst Adult Burn Patients Admitted to a Regional Burn Center: Twenty-Year Review. J. Burn Care Res..

[38] Alipour J., Mehdipour Y., Karimi A. (2020). Epidemiology and Outcome Analysis of 3030 Burn Patients with an ICD-10 Approach. Ann. Burn. Fire Disasters.

[39] Hung T.D., Lam N.N., Hung N.T. (2024). Prognostic Values of Neutrophil/Lymphocyte Ratio in Severe Burn Patients. Ann. Burn. Fire Disasters.

[40] Gharibeh T., Abu-Helalah M., Alshraideh H., Awwad M.A., Bzour Z.A., Abuzayed M., Taweel L., Al-Fayyadh Z., Wraikat B., Alfaqeeh Y. (2025). Predictors of Mortality in Medical ICU Patients: A Retrospective Study in a Tertiary Care Center in Jordan. J. Clin. Med..

[41] Rafla K., Tredget E.E. (2011). Infection Control in the Burn Unit. Burns.

[42] Forrester J.D., Berndtson A.E., Santorelli J., Raschke E., Weiser T.G., Coombs A.V., Sawyer R.G., Chou J., Knight H.P., Valenzuela J.Y. (2020). Survey of National Surgical Site Infection Surveillance Programs in Low- and Middle-Income Countries. Surg. Infect..

[43] Wild A., Shortall C., Dewachi O., Naim C., Green A., Hussain S., Abbara A. (2025). Conflict-Associated Wounds and Burns Infected with GLASS Pathogens in the Eastern Mediterranean Region: A Systematic Review. BMC Infect. Dis..

[44] Azzopardi E.A., Azzopardi E., Camilleri L., Villapalos J., Boyce D.E., Dziewulski P., Dickson W.A., Whitaker I.S. (2014). Gram Negative Wound Infection in Hospitalised Adult Burn Patients-Systematic Review and Metanalysis-. PLoS ONE.

